# Bacterial porphyrins in healthy skin: Microbiota components impact melanogenesis and age‐related processes leading to Porphyr'ageing

**DOI:** 10.1111/ics.70014

**Published:** 2025-09-10

**Authors:** Marie Meunier, Morgane De Tollenaere, Cyrille Jarrin, Emilie Chapuis, Marine Bracq, Laura Lapierre, Catherine Zanchetta, Jean Tiguemounine, Amandine Scandolera, Romain Reynaud

**Affiliations:** ^1^ Givaudan Active Beauty, Research and Development, Givaudan France SAS Argenteuil France; ^2^ Plastic Surgery, Polyclinique Courlancy Reims France

**Keywords:** hyperpigmentation, penetration, precursor spot, skin ageing, skin physiology/structure, statistics, TSPO/PBR, wrinkle

## Abstract

**Objective:**

Porphyrins are ubiquitous metabolites and are constitutive of the bacterial metabolome of healthy skin. Their consideration has until now been limited to their pro‐inflammatory activity in *acne vulgaris*. The present work suggests a new role for these molecules in the onset of skin ageing.

**Methods:**

A mixture of coproporphyrin III and protoporphyrin IX, representative of skin microbiota metabolites, was defined and applied in different skin models. Finally, an in vivo study was conducted to determine the association between porphyrin's abundance and ageing signs.

**Results:**

Bacterial porphyrins penetrated *stratum corneum* and reached living epidermal cells. The porphyrin mixture increased IL‐8, ROS and melanin contents. Porphyrin‐induced melanin synthesis appeared to be regulated by translocator protein TSPO/PBR. In fibroblasts, bacterial metabolites down‐regulated a set of transcripts involved in extracellular matrix architecture and associated with ageing, which was confirmed by a decrease of type I pro‐collagen. Finally, the clinical study established positive and significant correlations between porphyrin abundance and the severity of ageing signs, including invisible spots, brown spots and wrinkles length.

**Conclusion:**

In summary, porphyrins could play unexpected roles in skin's premature ageing process, a phenomenon we propose to call Porphyr'ageing, that promotes hyperpigmentation, inflammation, oxidative stress and fibroblast cell ageing, leading to dermal matrix weakening.

## Graphical abstract

Porphyrins, previously linked only to acne inflammation, are now implicated in skin ageing. Their presence correlates with increased melanin, inflammation, and oxidative stress, contributing to premature ageing signs. This study introduces the concept of “Porphyr'ageing,” highlighting the detrimental effects of bacterial metabolites on skin health and structure.

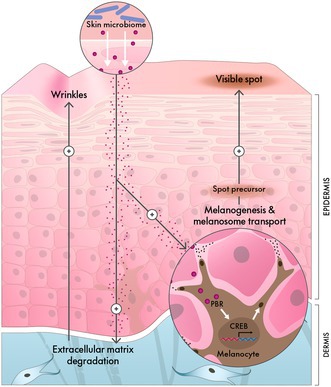

## Résumé

**Objectif:**

Les porphyrines sont des métabolites omniprésents et constituent le métabolome bactérien de la peau saine. Leur prise en compte a jusqu’à présent été limitée à leur activité pro‐inflammatoire dans l’acné vulgaire. Le présent travail suggère un nouveau rôle pour ces molécules dans l’apparition du vieillissement cutané.

**Méthodes:**

Un mélange de coproporphyrine III et de protoporphyrine IX, représentatif des métabolites du microbiote cutané, a été défini et appliqué dans différents modèles cutanés. Enfin, une étude in vivo a été réalisée pour déterminer l’association entre l’abondance des porphyrines et les signes de vieillissement.

**Résultats:**

Les porphyrines bactériennes ont pénétré dans la couche cornée et atteint les cellules épidermiques vivantes. Le mélange de porphyrines a augmenté les contenus en IL‐8, ROS et mélanine. La synthèse de mélanine induite par les porphyrines semble être régulée par la protéine translocatrice TSPO/PBR. Dans les fibroblastes, les métabolites bactériens ont régulé à la baisse un ensemble de transcrits impliqués dans l’architecture de la matrice extracellulaire et associés au vieillissement, ce qui a été confirmé par une diminution du pro‐collagène de type I. Enfin, l’étude clinique a établi des corrélations positives et significatives entre l’abondance des porphyrines et la gravité des signes de vieillissement, y compris les taches invisibles, les taches brunes et la longueur des rides.

**Conclusion:**

En résumé, les porphyrines pourraient jouer des rôles inattendus dans le processus de vieillissement prématuré de la peau, un phénomène que nous proposons d’appeler Porphyr’ageing, qui favorise l’hyperpigmentation, l’inflammation, le stress oxydatif et le vieillissement des cellules fibroblastes, conduisant à un affaiblissement de la matrice dermique.

## INTRODUCTION

Skin ageing results from the interplay between the chronological ageing, driven by genetic elements, and extrinsic factors, mostly ultraviolet rays (UV), that accelerate this process. Decline of DNA repairing process, decrease of cell proliferation and increase of cell senescence, reduced vascularization, disruption of barrier function and extracellular matrix integrity play key roles in the alteration of skin structure resulting in laxity, wrinkles, skin thinning, low hydration and pigmentation disorders [1, 2]. In the past decade, several works described changes in skin microbiota associated with ageing, and it emerged that bacteria could interfere in this process [3, 4, 5, 6]. The human skin hosts different types of microorganisms, including bacteria, fungi and viruses. Bacteria predominate microbiota with actinobacteria, firmicutes, proteobacteria and bacteroidetes being the major phyla detected on adult skin [7]. Their abundance depends on several parameters. The biogeography (body site) influences microbial communities at the appendage scale but also at a fine‐scale level, as example when comparing the different part of face [8, 9]. The skin of face is characterized by high sebum level and low microbial diversity compared to other body parts. In this site, microbiota is mainly composed of *Cutibacterium* sp., *Corynebacterium* sp. and *Staphylococcus* sp. [10, 11].

Porphyrins are ubiquitous intermediate metabolites of the heme, cobalamin and chlorophyll biosynthesis. In Gram‐negative bacteria and eukaryotes, including human beings, porphyrin biosynthesis follows the classical pathway using protoporphyrin IX (PPIX) as the final intermediate leading to heme [12, 13]. In Gram‐positive Firmicutes and Actinobacteria, including *Cutibacterium* and *Corynebacterium*, a non‐canonical coproporphyrin‐dependent pathway is in place. Porphyrins are fluorescent molecules observable at the skin surface under UV light, especially on the nose, chin, forehead and cheek, where follicles are more conspicuous [14, 15, 16]. While these molecules are detected on healthy skin, research efforts mainly focused on their association with *Cutibacterium acnes* and *acne vulgaris* or their antimicrobial effect using photoinactivation [17, 18, 19]. If *Cutibacterium* sp. is considered a major contributor to porphyrin levels in human skin, recent data suggest that other bacteria produce heme precursors [20]. Porphyrin's fluorescence was recently associated with *Staphylococcus epidermidis* [21]. Moreover, several skin bacteria, including *Bacilli*, *Proteobacteria*, *Corynebacteriales* and *Micrococcales*, are predicted to possess the genes encoding for porphyrin metabolism or were shown to produce those compounds [22, 23, 24, 25, 26].

The impact of bacterial porphyrins in skin physiology remains unclear. The extensive literature on *acne vulgaris* pointed out that porphyrins secreted by *C. acnes*, especially CPIII, have pro‐inflammatory activity [18, 19, 27, 28, 29]. However, *Cutibacterium* has also to be viewed as a skin commensal, contributing to the balance of microbiota, and porphyrins were recently proven to shape bacterial community composition in human skin [6, 20, 30, 31]. Interestingly, a porphyrin used clinically to treat skin diseases was demonstrated to stimulate melanogenesis, questioning their possible role in host metabolism [32].

In this study, we aimed at identifying the biological pathways associated with the interaction between porphyrins and skin cells. Their ability to penetrate skin was addressed ex vivo. A mixture of porphyrin representative of those synthesized by the microbiota of the face was defined, and its pro‐inflammatory and pro‐pigmenting activities were evaluated in co‐cultures of keratinocytes and melanocytes. The impact of this mixture was also observed in fibroblasts through a gene expression analysis and type I pro‐collagen quantification. Finally, a correlative clinical study involving 100 volunteers was conducted to determine the relationship between porphyrin abundance and signs of ageing, including invisible spots, brown spots and wrinkle length.

## MATERIALS AND METHODS

### Chemicals

CPIII and PPIX were supplied from Fischer Scientific and Enzo Life Science, respectively. The mixture contained a ratio of CPIII and PPIX of 100:1, with CPIII at 10 μM and PPIX at 0.1 μM, and was first diluted in DMSO (dimethylsulphoxide) and then in suitable culture medium. As porphyrins are photosensitive components, solutions and treated biological samples were protected from light.

### Skin penetration analysis by Raman spectroscopy

#### Skin explant culture and treatment

Skin explants were obtained with the informed consent from breast surgeries of a 57‐year‐old female donor. The tissue was kept alive by culturing on biocompatible plastic grids in standard 24‐well plates in an air–liquid interface with skin culture medium (Givaudan) at 37°C with 5% CO_2_. The culture medium was renewed every 24 h. Skin explant was topically treated with CPIII at 100 μM for 8 h. The untreated condition did not receive any treatment. After the end of incubation, the skin surface was cleaned to eliminate any excess of the product. The skin explants were then frozen at −80°C and cut longitudinally using a cryotome with a thickness of 20 μm. Three tissue sections were selected and deposited on a CaF2 support for Raman imaging analysis for a total of 6 Raman images per condition.

#### Raman micro‐imaging

Raman images have a size of Y: 10 μm/X: 120 μm with a step of 5 μm in X and 5 μm in Y. Each Raman image has 3Y spectra and 23X spectra (69 spectra per image). Conditions of analysis were as follows: laser wavelength: 660 nm; objective: 100 X, long focal length with a numerical aperture of 0.75; acquisition time: 20 seconds; accumulation: 1X; spectral range: 400 to 3100 cm^−1^; grating: 1200 T; confocal hole: 500 μm; slit width: 100 μm (spectral resolution 6.5 cm^−1^) and step in X/Y: 5 μm. In order to ensure reproducibility of the measurements before each use, the Raman spectrometer is calibrated with silicon which gives a Raman peak at 520.7 cm^−1^. Continuous control of the laser power at the sample level is achieved.

A pre‐processing of Raman images was made by eliminating aberrant spectra (fluorescence, burning, saturation), correcting the baseline, applying a spectral smoothing and despiking, and a spectral normalization. After data pre‐processing, the parameter of skin porphyrin markers was calculated on the overall spectral image to determine the penetration and distribution of porphyrin in the skin section. The processing of corrected data maps was performed by using homemade software that operates in the MATLAB environment. For the assessment of the porphyrin skin penetration, we calculated the integrated intensity of the spectral range 715–745 nm. This spectral region reflects the porphyrin fluorescence emission with 660 nm laser excitation and is associated with the degree of porphyrin penetration in the skin section. The reconstructed spectral images based on the integrated intensity of porphyrin fluorescence permit us to see the level of permeation and spatial distribution of porphyrin in the skin section up to 120 μm. The results were averaged over 3 measurements (*n* = 3) performed on different but adjacent skin sections.

### Study of bacterial porphyrin effect on melanin synthesis in keratinocyte/melanocyte co‐culture

#### Keratinocyte and melanocyte co‐culture and treatment

Normal human keratinocytes (NHKs) isolated from biopsies were seeded at 100000 cells per well in 6‐well plates pre‐coated with collagen I and, 24 h later, normal human melanocytes (NHMs) were seeded at 90000 cells per well. Cells were incubated for 48 h in complete medium (Dermalife medium supplemented with Life factors K) and then were stimulated for 3 days with porphyrin mixture (10 μM CPIII/0.1 μM PPIX) and dilutions (D2: 5 μM/0.05 μM; D10: 1 μM/0.01 μM) (*n* = 3). The treatments were diluted in DMSO and then in basal medium without supplements (Dermalife medium supplemented with 1% antibiotics).

#### Study of PBR inhibitor effect on porphyrin‐induced melanin synthesis

The cell culture was realized as described above. NHKs were seeded at 80000 cells per well and NHMs at 110 000 cells per well. After 48 h of incubation in complete medium, cells were stimulated for 4 days by porphyrin mixture (CPIII 10 μM/PPIX 0.1 μM) or combined with benzodiazepine receptor antagonist PK11195 (Abcam) at 75 μM (*n* = 3). Before dilution in basal medium (Dermalife supplement with 1% antibiotics), porphyrin mixture was diluted in DMSO and PK11195 in absolute ethanol. Treatments were renewed every 2 days.

#### Melanin extraction and dosage

After treatment, cells were rinsed off with PBS (phosphate buffer saline), and 200 μL of a NaOH 0.5 N solution was added in each well. The cell lysates were collected. In parallel, a standard range of 8 concentrations of melanin was prepared (0 to 100 μg.mL^−1^). Samples were heated for 1 h at 80°C. Each sample (100 μL) was transferred to a 96‐well plate, and optical density was measured at 405 nm with a microplate reader (Spark®, TECAN).

### Study of bacterial porphyrin effect on Interleukin‐8 production in keratinocyte/melanocyte co‐culture

#### Keratinocyte and melanocyte co‐culture and treatment

NHKs and NHMs co‐culture was established as previously described and cells were stimulated for 3 days with the porphyrin mixture (CPIII 10 μM/PPIX 0.1 μM) (*n* = 3). Treatments were diluted in DMSO, then in basal medium without supplements (Dermalife medium supplemented with 1% antibiotics), were renewed every day, and supernatants were collected after 2 days of culture.

#### Interleukin‐8 quantification

Interleukin 8 (IL‐8) quantification was realized with the Human IL‐8/CXCL8 Quantikine ELISA kit (D8000C, R&D Systems) according to the manufacturer's instructions.

### Study of bacterial porphyrin impact on oxidative stress in keratinocytes

#### Cell culture and treatment

NHKs isolated from biopsies were seeded in a black plate with a glass bottom, pre‐coated with collagen I at 20 000 cells per well, in *n* = 4. Cells were incubated for 48 h in complete medium (Dermalife medium supplemented with Life factors K) at 37°C with 5% CO_2_.

#### Treatment and quantification of reactive oxygen species

After incubation, the 2′,7′‐Dichlorofluorescin diacetate (DCFH‐DA, Sigma) probe was added at 50 μM for at least 60 min at 37°C. Cells were then washed two times with PBS buffer and treated with the following conditions: porphyrin mixture (CPIII 10 μM, PPIX 0.1 μM) or tert‐Butyl hydroperoxide solution (TBP, Sigma) at 5 mM, used as a positive pro‐oxidant reference. Untreated cells remained in PBS. ROS concentration was determined by measuring fluorescence in darkness (excitation wavelength: 488 nm; emission wavelength: 525 nm) with a microplate reader (Spark®, TECAN).

### Study of bacterial porphyrin effect on NHDFs through gene expression analysis

Normal Human Dermal Fibroblasts (NHDFs) were seeded at 300 000 cells per well in a 6‐well plate. After 48 h of incubation, the cells were rinsed with PBS and put in resting overnight in DMEM medium without foetal bovine serum (FBS). The cells were stimulated with the representative mixture of porphyrins CPIII (10 μM) and PPIX (0.1 μM). The treatments were diluted in DMSO, then in basal medium without supplements (Dermalife medium supplemented with 1% antibiotics). After 6 h of stimulation, total RNAs were extracted by the TRIzol method, and after retrotranscription, gene expression was evaluated by quantitative PCR (see details in Supplementary Material S3).

### Study of porphyrin mixture effect on pro‐collagen I synthesis in fibroblasts

#### Cell culture and treatment

NHDFs isolated from biopsies were seeded in a 6‐well plate at 100 000 cells per well in triplicate. Cells were incubated for 48 h in complete DMEM medium supplemented with 10% FBS and 1% antibiotics at 37°C with 5% CO_2_. Then, cells were rinsed twice with PBS and cultured in basal medium (DMEM without FBS) with 1% antibiotics that were supplemented with porphyrin mixture (CPIII 10 μM, PPIX 0.1 μM) or with porphyrin mixture 10X (CPIII 100 μM, PPIX 1 μM). Cells were incubated for 48 h at 37°C, 5% CO_2_. NHDFs cultivated in basal medium with 1% antibiotics were used as a negative control. Finally, media were collected, centrifuged at 2000×*g* for 10 min at 4°C and stored at −20°C until pro‐collagen I quantification.

#### Type I pro‐collagen quantification

Type I pro‐collagen was quantified using the Human Pro‐Collagen I alpha 1 ELISA Kit (ab210966, Abcam) according to the manufacturer's instructions. Supernatants were diluted at 1:200 in sample diluent.

### Quantification of bacterial porphyrins, wrinkle length, precursor spots and brown spots in vivo

The clinical study was conducted in France, on 100 volunteers (78 women and 22 men) aged between 18 and 64 years. Participants with skin issues were excluded from the study. The phototypes of the participants ranged from 1 to 3 on the Fitzpatrick scale. The study took place during the spring season to minimize changes in melanin content and microbiome composition and behaviour due to high UV exposure. All the subjects participating gave their informed consent, signed at the beginning of the study. The study followed and was in compliance with the tenets of the Declaration of Helsinki.

Porphyrin intensity and count, average wrinkle length, precursor/invisible spots count (i.e. invisible spots detected under specific lighting modality) and brown spots count (also known as age spots or hyperpigmented spots) were quantified using Visia®‐CR 2.3 (Canfield Scientific, Parsippany, NJ, USA). According to the VISIA® constructor, porphyrins were visualized under red lighting modality, invisible spots under blue light excitation and brown spots under cross‐polarized lighting. Participants underwent a 15‐min acclimatization period in an environment maintained at 20 to 24°C and 40% to 60% humidity. Precursor spots, brown spots, porphyrin levels and porphyrin intensity were assessed using Vaestro™ software, and data were normalized to the skin surface analysed.

Linear regression and Pearson's correlation analyses were performed to evaluate significant correlations between parameters. *p*‐values <0.05 were considered indicative of significant correlations. Graphics, correlation coefficient calculation and regression line equation were determined using the software R version 4.4.0 (R core Team, 2021), with the packages ggplot2 [33] and rstatix [34].

### Statistical analysis

A Shapiro–Wilk normality test has been performed to evaluate whether the data follow the Gaussian law. If results did not follow the Gaussian law, a non‐parametric statistical analysis was performed by Kruskal–Wallis ANOVA followed by Mann–Whitney *U* test. When results followed the Gaussian law, a parametric Students' *t*‐test was applied. It was considered a significant result with #*p* < 0.1, *p* < 0.05 with *, *p* < 0.01 with ** and *p* < 0.001 with ***.

## RESULTS AND DISCUSSION

### A porphyrin penetrates *stratum corneum* and reaches epidermal cells from the basal layer

**FIGURE 1:**
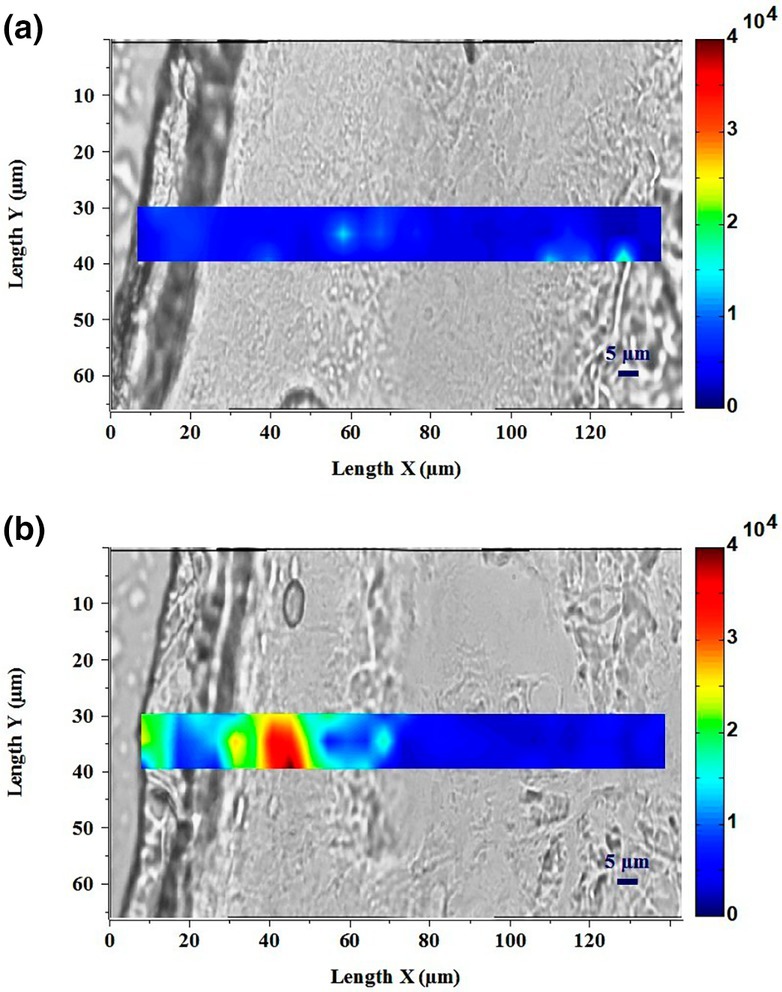
Skin sections and profile analysis using Raman spectroscopy of skin explants with (a) no treatment (control) and (b) 8 h after topical treatment with coproporphyrin III (100 μM). Porphyrin relative concentration is indicated by colour scale from dark blue (low concentration) to dark red (high concentration) expressed in arbitrary unit and materialize molecule penetration.

To determine the biological significance of the interaction between skin cells and tetrapyrrole molecules, a major bacterial porphyrin identified in the cutaneous tissue, was topically applied on human skin explants. Its penetration was observed thanks to porphyrin's fluorescence observed by Raman spectroscopy imaging (Figure 1). Low fluorescence background signal specific to constitutive porphyrins was detected in the section of untreated explants. In the treated explants, the fluorescence signal specific to porphyrins was detected in the epidermis up to 65 μm and the maximum signal was registered between 25 and 50 μm of depth. CPIII was able to fully cross *stratum corneum*, reported to vary from 11 to 18.3 μm, and molecules concentrated in the epidermis [35]. The mean epidermal thickness of the cheek, being 78.8 ± 24.4 μm, is very close to values reported for breast tissue, at 76.9 ± 26.2 μm [36, 37]. These data make it reasonable to conclude that porphyrins can reach living cells of the epidermis, including melanocytes and keratinocytes, and are susceptible to attain the dermal–epidermal junction.

### Definition of bacterial porphyrin composition

A mixture representative of the metabolites excreted by human skin microbiota, outside pathological conditions, was defined based on data available in the literature. In vivo, CPIII is the major porphyrin identified in the skin of healthy and acne patients [18, 29, 38]. PPIX was detected on healthy skin and determined as a minor element [27, 39, 40]. Based on these data, a representative mixture of skin microbiota porphyrins would contain CPIII as the main component and PPIX at a lower concentration.

Little is known on the concentration of these metabolites in healthy skin making it difficult to determine physiologically relevant dosage. In vivo, CPIII was identified as the main component of comedo contents, reaching 0.03 to 3.18 ng/mg^−1^ [27, 29]. In vitro, the concentration of total porphyrins in *C. acnes* culture media can reach 200 pmol/mg dry cells or 20 μM and varies depending on strains [28, 41, 42, 43]. The proportion of porphyrins in *C. acnes* cells grown in vitro was determined as CPIII and its methyl ester at 99% and PPIX at 1% [19]. Coproporphyrin also predominates in other bacteria inhabiting human skin, such as *Staphylococcus* sp. or *Corynebacterium* sp., with concentrations in culture media varying from 0.94 to 2.3 μM [17, 44, 45].

Previous in vitro studies on keratinocytes and sebocytes used CPIII or porphyrin mixture from 2.5 to 40 μM [19, 29]. Furthermore, PPIX exhibited no cytotoxic effects on melanocytes below 30 μM [32].

The cytotoxicity was here evaluated on normal human keratinocytes (NHKs) and normal human melanocytes (NHMs) (Data S1). CPIII, in the range 7.6 μM‐1.5 mM, displayed no cytotoxic effect neither on keratinocytes at 24 h or 48 h, nor in melanocytes at 24 h, but slightly affected their cell viability at 48 h, around 70%. PPIX affected cell viability at lower concentrations. Cytotoxicity was higher at 24 h compared to the 48 h time point. Cell viability at 24 h remained greater than 70% until 8.9 μM in melanocytes and until 53.3 μM in keratinocytes. Cell viability at 48 h remained greater than 80% until 8.9 μM in melanocytes and until 88.9 μM in keratinocytes. Therefore, the concentration of CPIII was determined at 10 μM, being in the same range as previous studies, and PPIX was set at 0.1 μM according to the ratio determined by Spittaels et al. Then, this determined ratio was tested on a co‐culture of normal human melanocytes and keratinocytes treated for 72 h, and a non‐cytotoxic effect was observed (Data S1).

### Dose‐response pro‐melanogenic activity of bacterial porphyrins is controlled by TSPO/PBR

**FIGURE 2:**
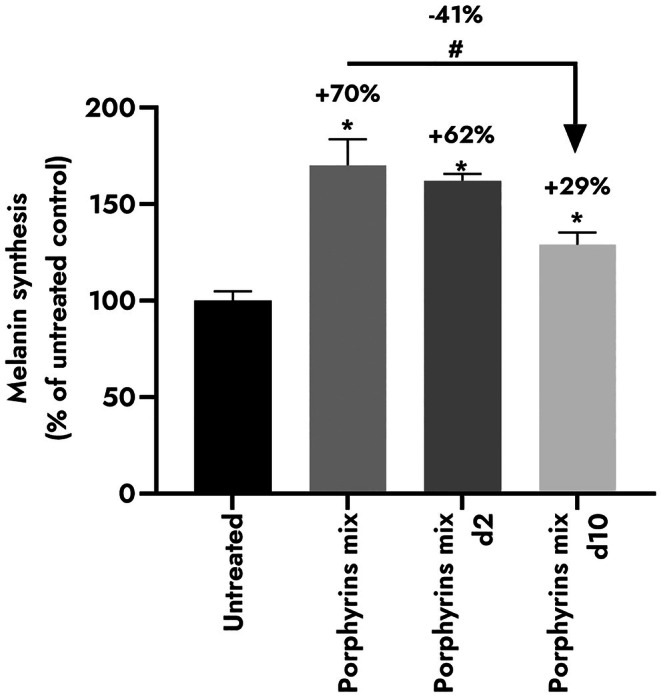
Quantification of melanin extracted from a keratinocyte/melanocyte co‐culture (*n* = 3) depending on the concentration of porphyrin mixture: Coproporphyrin III (CPIII) 10 μM and protoporphyrin IX (PPIX) 0.1 μM; d2: CPIII 5 μM, PPIX 0.05 μM; d10: CPIII 1 μM, PPIX 0.01 μM. Results are expressed in % of control condition ±SEM. Statistics determined by Mann–Whitney test with #*p* < 0.1, *p < 0.05.

Previous studies reported that PPIX stimulates melanin synthesis and melanosome transport [32, 46]. The co‐culture of keratinocytes and melanocytes was treated with a porphyrin mixture at native concentration and diluted twice and 10 times (Figure 2). A dose‐response pro‐melanogenic activity of the porphyrin mixture was observed: melanin content increased by +70% with CPIII 10 μM and PPIX 0.1 μM; by +62% with CPIII 5 μM and PPIX 0.05 μM; and by +29% using CPIII 1 μM and PPIX 0.01 μM. We confirmed that bacterial porphyrins promote melanogenesis in human melanocytes. Our results are in line with a previous study that established *C. acnes* increases melanin content in melanocytes and could be responsible for acne‐related post‐inflammatory hyperpigmentation [47]. Taken together, these findings suggest that the microbiota could influence skin pigmentation through porphyrins.

The TSPO (translocator protein) formerly peripheral benzodiazepine receptor (PBR) is a mitochondrial receptor, highly conserved among species and a structural homologue of the mammalian TSPO/PBR has been identified in *Bacillus cereus* that regulates intracellular levels of metabolites from the coproporphyrin‐dependent heme synthesis pathway [48].

In human skin, the receptor is strongly expressed where it may play a protective role against free radicals (ROS) and regulate UV‐induced apoptosis [49, 50]. TSPO/PBR was reported to be involved in melanogenesis regulation when activated by diazepam [51]. CPIII and PPIX have been reported as ligands of TSPO/PBR, facilitating the intracellular transport of heme intermediates [52, 53]. Nevertheless, the link between porphyrin‐induced melanogenesis and TSPO/PBR has never been explored.

**FIGURE 3:**
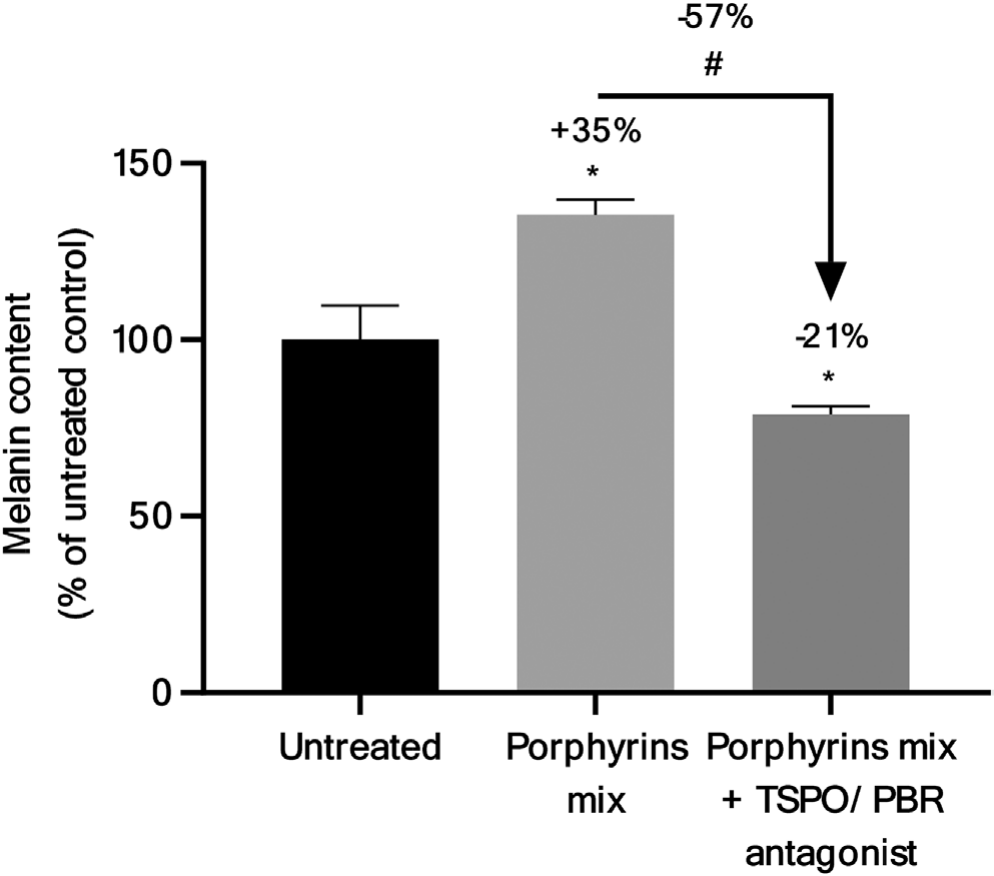
Quantification of melanin after treatment of keratinocyte/melanocyte co‐culture (*n* = 3) with the mixture of porphyrins (coproporphyrin III 10 μM and protoporphyrin IX 0.1 μM) in absence or presence of translocator protein (TSPO/PBR) antagonist. Results are expressed in % of control condition ±SEM. Statistics determined by Mann–Whitney test with #*p* < 0.1 and *p < 0.05.

Melanin synthesis was monitored in the presence of porphyrin mixture alone or combined with TSPO/PBR antagonist PK11195, an isoquinoline carboxamide derivative binding the receptor with high affinity and selectivity [54]. TSPO/PBR antagonist (75 μM) significantly decreased melanin synthesis induced by porphyrin mixture (Figure 3). These results are aligned with the ability of porphyrins to fully reach the living cells of the epidermis, where TSPO/PBR is expressed, as demonstrated during Raman imaging experiments. This work established for the first time that TSPO/PBR is involved in the melanogenesis signalling pathway triggered by bacterial porphyrins.

### Bacterial porphyrins are pro‐inflammatory compounds and increase oxidative stress

**FIGURE 4:**
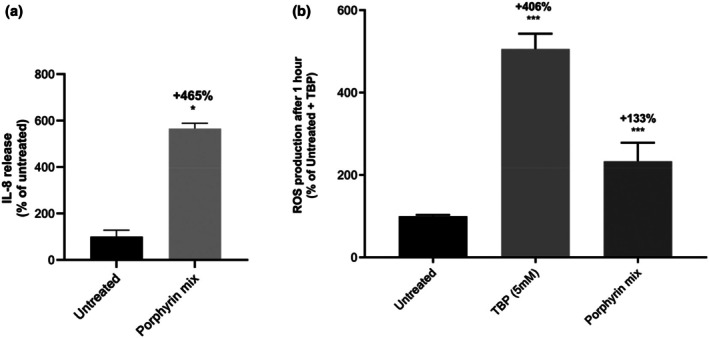
Effect of the porphyrin mixture (coproporphyrin III 10 μM and protoporphyrin IX 0.1 μM) on (a) the release of IL‐8 in the supernatant of a keratinocyte/melanocyte co‐culture untreated or after stress induced with porphyrins, (b) reactive oxygen species (ROS) production in normal human keratinocytes after 1 h treatment with pro‐oxidant TBP (tert‐Butyl hydroperoxide solution, 5 mM) as positive reference or with porphyrins. Results are expressed in % of control condition ±SEM. Statistics determined by Mann–Whitney test with *p < 0.05 and ***p < 0.001.

Oxidative stress and inflammation are elements of the immune response and are involved in skin ageing and pigmentation [1, 55]. The pro‐inflammatory activity of bacterial porphyrins was previously reported in the context of acne, so we aimed to confirm these results and evaluate their impact on ROS production in skin cells [19, 29]. NHKs and NHMs were treated with the porphyrin mixture (CPIII 10 μM and PPIX 0.1 μM), and the release of pro‐inflammatory chemokine IL‐8 was quantified in culture medium (Figure 4a). Compared to untreated cells, the porphyrin mixture induced IL‐8 release by +465% in the culture medium. Our results are consistent with previous findings showing that CPIII tetramethyl ester increases IL‐8 and TNFα (tumour necrosis factor α) mRNA expression in keratinocytes and melanocytes and that porphyrins isolated from *Cutibacterium* induce IL‐1β and NLRP3 inflammasome [19, 29, 47]. IL‐8, which is activated by interleukin IL1‐β / TNFα (tumour necrosis factor α)/NF‐κB (nuclear factor κB) inflammatory pathway, is part of skin ageing–associated secreted proteins and is a biomarker of inflammageing: a process where cell senescence is driven by chronic inflammation and oxidative stress caused by sun exposure and all other environmental stressors skin is subjected to [55, 56, 57, 58, 59].

Intracellular ROS content was evaluated in keratinocytes treated with porphyrin mixture (Figure 4b). TBP was used as a positive control and increased ROS content in normal human keratinocytes by +406%. Bacterial porphyrins did increase ROS content in keratinocytes by +133%.

Oxidative stress is known to trigger melanin synthesis, which is an oxygen‐dependent process, and matrix degrading enzymes that contribute to premature ageing signs, such as wrinkles and hyperpigmentation [60, 61]. It causes DNA damage and telomere shortening, involved in cell senescence, another key feature of skin ageing [1].

### Bacterial porphyrin impact fibroblast gene's expression and down‐regulates type I pro‐collagen I

According to the Raman spectroscopy imaging experiment, we concluded that the interaction of porphyrins with dermal cells is a likely hypothesis. A gene expression analysis was conducted on NHDFs with the porphyrin mixture (Table 1). The expression of *COL1A1* was down‐regulated together with 37 other transcripts, with two genes strongly repressed: *NTF3* and *TERT* encoding the telomerase reverse transcriptase. The list of genes is described in Data S2, which reviews more precisely the literature establishing a link between 30 genes studied and ageing or senescence.

**TABLE 1:** Expression fold change of 38 genes involved in different function (bold names on the left) in normal human fibroblasts treated with porphyrin mixture (coproporphyrin III 10 μM and protoporphyrin IX 0.1 μM, *n* = 3), relative to control.

Gene name	Fold change	SD	*p*‐value	Pathway
**VEGFA**	−1.74	0.110	0.005532	Angiogenesis
**FGF2**	−1.87	0.053	0.000264	Proliferation/apoptosis
**IGFBP3**	−1.47	0.043	0.000527	
**COL7A1**	−1.68	0.096	0.003654	DEJ
**COL4A1**	−1.31	0.054	0.003244	
**DDIT3**	−2.04	0.033	0.000025	DNA repair
**GADD45A**	−1.68	0.0146	0.000005	
**XPC**	−1.55	0.072	0.00208	
**SOD2**	−1.34	0.083	0.01135	Enzymatic antioxidant defence
**GPX1**	−1.34	0.023	0.000064	
**CYR61**	−2.38	0.101	0.001297	Extracellular matrix
**CTGF**	−1.90	0.0388	0.000059	
**FBN2**	−1.55	0.0292	0.000043	
**FBN1**	−1.52	0.0193	0.000024	
**HPSE**	−1.47	0.0280	0.000104	
**HSPG2**	−1.44	0.0813	0.005874	
**TIMP2**	−1.43	0.0039	<0.000001	
**MMP3**	−1.40	0.0704	0.004733	
**FBLN5**	−1.36	0.0972	0.019899	
**ELN**	−1.35	0.1023	0.022646	
**SPARC**	−1.35	0.0300	0.000224	
**FMOD**	−1.33	0.0435	0.001474	
**COL1A1**	−1.33	0.1170	0.035355	
**SDC1**	−1.32	0.0021	<0.000001	
**LOXL2**	−1.30	0.0663	0.008318	
**VCAN**	−1.30	0.0051	<0.000001	
**TGFB1**	−1.54	0.0395	0.000205	Fibrosis
**SIRT2**	−1.47	0.0139	0.000004	Gene silencing
**RORA**	−1.69	0.0602	0.000768	Melatonin receptor
**NQO2**	−1.35	0.0110	0.000003	
**NTF3**	−3.09	0.0539	0.000057	Neurotrophin
**NGF**	−1.84	0.0107	<0.000001	
**BDNF**	−1.50	0.0526	0.000891	
**SIRT1**	−1.39	0.0607	0.002465	p53 regulator
**NANOG**	−1.70	0.0645	0.000722	Pluripotency transcription factor
**POU5F1**	−1.49	0.0420	0.000357	
**TERT**	−5.50	0.2573	0.011207	Telomere maintenance
**MYC**	−1.50	0.0660	0.001974	Transcription factor

*Note*: Statistical analysis with Students' *t*‐test. Genes nomenclature available in Data S2.

Abbreviation: DEJ, dermal–epidermal junction.

The decrease of type I pro‐collagen synthesis is a trait of chronological and photo‐ageing, leading to extracellular matrix weakening and attributable to *COL1A1* down‐regulation [62, 63, 64]. The down‐regulation of other transcripts suggested deleterious effects of porphyrins on dermal matrix and skin architecture: mRNA encoding matrix constituents (elastin, fibulin, fibrillin, fibromodulin, heparan sulphate proteoglycan, syndecan and versican) and dermal‐epidermal junction constituents (collagen IV, collagen VII); regulators of matrix homeostasis (*FGF2*, *LOXL2*, *SPARC*, *TIMP2*) and of cell proliferation (*IGFBP3*, *TGFB1*).

*TERT* encodes the telomerase reverse transcriptase that controls the shortening of telomeric ends happening with every cell division, and the decrease of enzyme activity is associated with senescence and ageing [56, 65, 66, 67].

*NTF3* encodes neurotrophin‐3 that belongs to a family of growth factors including brain‐derived neurotrophic factor (BDNF) and nerve growth factor (NGF), the three being down‐regulated by the CPIII/PPIX mixture. During ageing, NTF3 and NGF protein expression decreases, possibly contributing to inflammatory condition [68, 69]. While their exact role in this process was not explored, NTF3 increase in HaCat human keratinocytes leads to a decrease of interleukin 8, suggesting an anti‐inflammatory property [70]. Other transcripts slightly down‐regulated by bacterial porphyrins were found to be involved in ageing and inflammation. As an example, *SIRT1*, involved in longevity, and *SOD2* were down‐regulated by porphyrins that were observed above to promote IL‐8 and ROS. Under oxidative stress, SIRT1 increases SOD2 levels to prevent ROS production and thus limit synthesis of pro‐inflammatory cytokines such as IL‐8 [71]. A decreased expression of *SIRT1* might thus be related to *SOD2* down‐regulation and ROS/IL‐8 increase.

**FIGURE 5:**
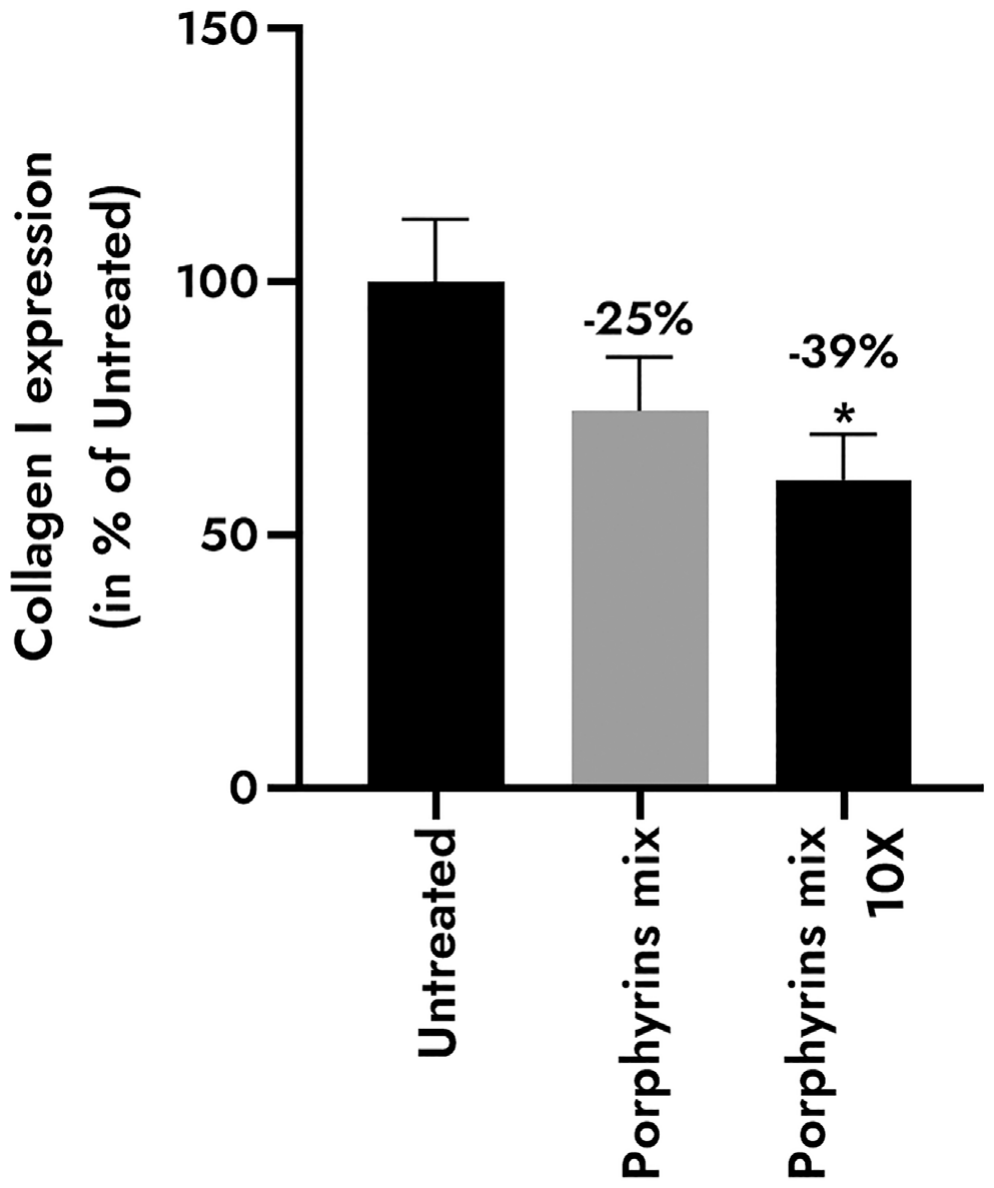
Type I pro‐collagen synthesis in normal human dermal fibroblasts depending on the concentration of porphyrin mixture: Coproporphyrin III (CPIII) 10 μM and protoporphyrin IX (PPIX) 0.1 μM; 10X: CPIII 100 μM, PPIX 1 μM. Results are expressed in % of control condition ±SEM. Statistics determined by Mann–Whitney test with *p < 0.05.

To confirm that the down‐regulation of COL1A1 impacts type I pro‐collagen synthesis, fibroblasts were exposed to bacterial porphyrins and the structural protein was quantified (Figure 5). The protein content decreased non‐significantly by −25% in NHDFs treated with the porphyrin mix, and significantly declined by −39% using the 10X porphyrin mix.

The down‐regulation of 30 genes indicated that bacterial porphyrins induce a phenotype profile of aged fibroblasts, consistent with the drop of type I pro‐collagen protein and *COL1A1* expression. This is the first report that bacterial porphyrins induce cellular senescence. Further studies will be necessary to confirm the down‐regulation at the protein level and decipher the exact role of porphyrins as the decrease of some transcripts (*GADD45A*, *CYR61* and *MMP3*) seems associated with protective effects.

### In vivo bacterial porphyrin intensity is correlated with the severity of ageing signs, including precursor and brown spots count and wrinkle length

**FIGURE 6:**
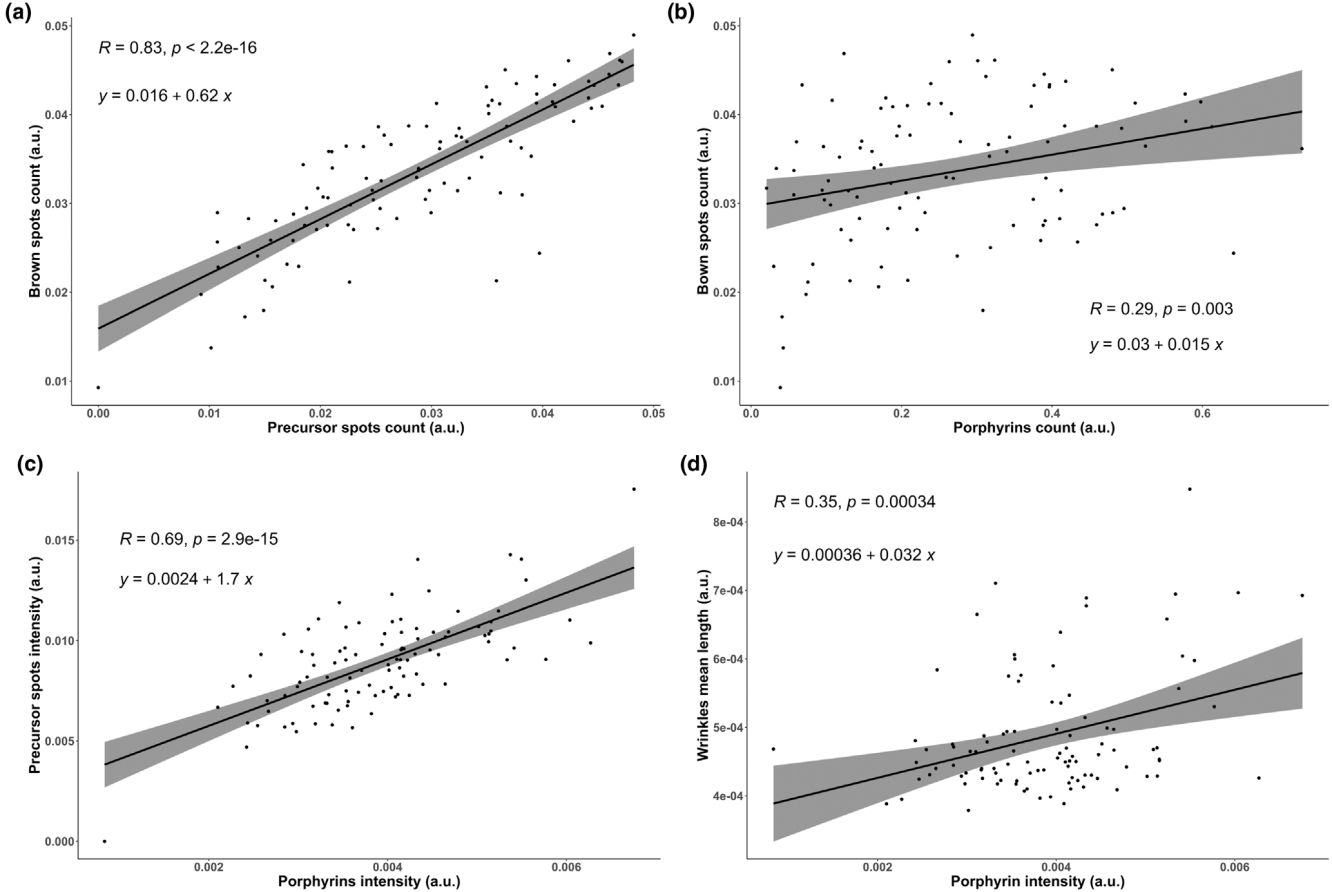
Linear regression analysis of clinical parameters determined on 100 volunteers presenting healthy skin: (a) normalized brown spots and normalized precursor spots counts; (b) normalized brown spots area and normalized porphyrin count; (c) normalized precursor spots and normalized porphyrin intensity; (d) normalized wrinkle mean length and normalized porphyrin intensity. *R* represents the Bravais–Pearson correlation coefficient, while *p* denotes the *p*‐value from the *t*‐test comparing *R* values to zero. A *p*‐value <0.05 indicates a significant linear relationship between the considered variables.

A clinical study was conducted on 100 volunteers (78 women and 22 men) from 18 to 64 years old, presenting healthy skin. Porphyrin intensity and count, average wrinkle length, invisible spots count (detected under blue light) and brown spots count (also known as age spots or hyperpigmented spots) were quantified using Visia®‐CR 2.3. Porphyrin's fluorescence can be considered as the reflect of porphyrins produced by the skin microbiota [18, 40]. Linear regressions were performed to analyse the relationships between porphyrin abundance‐related values and the other parameters, but also between invisible and brown spots. Results are presented in Figure 6. In skin, melanin produced by melanocytes accumulates mainly in the basal layer of epidermis and is transferred to keratinocytes via melanosomes [72, 73]. As the keratinocytes undergo differentiation and ascend, melanin is partially degraded and deposited within epidermal structure. In this study, invisible spots were observed to strongly correlate with brown spots (*R* = 0.83, *p* < 2.2.10^−16^), suggesting the first one being the precursor of the second, in line with the processus of melanin migration within epidermis. So, invisible spots were called precursor spots. Regardless of age, a significant and high correlation (*R* = 0.69, *p* < 2.9.10^−15^) between porphyrin intensity and precursor spots was revealed. A weak but significant association of porphyrin intensity was also observed with brown spots count (*R* = 0.29, *p* = 0.003) and mean of the wrinkle length (*R* = 0.35, *p* = 0.00034). Wellen et al. also reported a positive correlation between UV‐induced skin damage and facial porphyrin level in 92 subjects from 15 to 18 years [74].

Appearance of brown spots and pigment accumulation is related to skin cell senescence, inflammation and chronic sun exposure [75, 76]. Porphyrins have been previously reported to be responsible for oxidative stress in human facial skin [77]. Indeed, porphyrins are photosensitizers, excited by UV to high‐energy states, releasing singlet oxygen and leading to lipid peroxidation and protein and nucleic acid oxidation [78]. These results also confirm porphyrins to be pro‐inflammatory and pro‐oxidant.

Our in vitro experiments indicated that porphyrins stimulated melanin and decreased type I collagen synthesis in association with the gene expression of several transcripts involved in dermal matrix homeostasis, which contributed to accelerate fibroblast cell ageing. The correlations established at the clinical level between porphyrins and the severity of ageing signs, including brown spots or wrinkles, support these findings. Clinical data can be related to previous works revealing that *Cutibacterium*, considered a main producer of porphyrins, is associated with older‐looking skin in young individuals and tends to increase in hyperpigmented areas of the skin [22, 79].

## CONCLUSION

This study demonstrated the role of porphyrins produced by the skin microbiota in skin ageing, extending beyond their previously recognized function in acne. Porphyrins were found to penetrate skin up to the epidermis with possible interaction with dermal cells. Bacterial porphyrins triggered pro‐inflammatory cytokine, stimulated melanogenesis and displayed pro‐ageing effects on fibroblasts. Positive correlations between porphyrins and the severity of ageing signs like invisible spots, brown spots and wrinkle length were established from a cohort of 100 individuals, strengthening the hypothesis that bacterial metabolites contribute not only to inflammatory skin conditions, but could also participate in the early onset of visible signs of ageing—a phenomenon that we propose to call Porphyr'ageing. Understanding porphyrin metabolism in healthy skin and interactions with human cells will help to describe the fine‐tuning of skin homeostasis that microbiota and host orchestrate.

## AUTHOR CONTRIBUTIONS

**Marie Meunier and Amandine Scandolera:** Conceptualization. **Marie Meunier, Cyrille Jarrin, Emilie Chapuis and Morgane De Tollenaere:** Data curation and formal analysis. **Romain Reynaud:** Funding acquisition and resources. **Marie Meunier and Marine Bracq:** Investigation. **Marie Meunier, Cyrille Jarrin and Catherine Zanchetta:** Methodology. **Amandine Scandolera:** Project administration and supervision. **Marie Meunier, Morgane De Tollenaere, Catherine Zanchetta and Amandine Scandolera:** Verification. **Morgane De Tollenaere and Catherine Zanchetta:** Writing review and editing.

## CONFLICT OF INTEREST STATEMENT

Marie Meunier, Morgane De Tollenaere, Cyrille Jarrin, Emilie Chapuis, Marine Bracq, Laura Lapierre, Catherine Zanchetta, Amandine Scandolera and Romain Reynaud were employed by the company Givaudan Active Beauty.

## ETHICS STATEMENT

The study was conducted according to the guidelines of the Declaration of Helsinki in effect in 2017, and written informed consent was obtained from all subjects involved in the study. The volunteers were informed of their right to object and were informed of the measures in place for the protection of their personal data. This research does not involve human subjects (Loi Jardé, text no. 2012‐300 of March 5, 2012) and is therefore not subject to ethical evaluation by a CPP (Comité de Protection des Personnes) according to French law. This study was conducted in 2017, before the obligation of an IRB review, and no IRB review can be issued retrospectively according to Givaudan's Principles of Conduct.

## Supporting information


Data S1:

Data S2:

Data S3:

## Data Availability

The data that support the findings of this study are available from the corresponding author upon reasonable request.
