# Supplementary material for: Bacterial porphyrins in healthy skin: Microbiota components impact melanogenesis and age‐related processes leading to Porphyr'ageing
Source: Int J Cosmet Sci. 2025 Sep 10;48(1):186–99. doi: 10.1111/ics.70014 (PMC12877991; doi:10.1111/ics.70014)
Supplement: Supplementary file 1 — Data S1: [file ICS-48-186-s002.docx]

**Supplementary data S1**. Cytotoxicity assays on normal human (**a**) melanocytes and (**b**) keratinocytes, using MTT assay, after 24 h or 48 h of treatment with coproporphyrin III (7.6 µM-1.5 mM) and protoporphyrin IX (0.1 µM-1.8 mM). **(e)** Cytotoxicity assay of porphyrin mix (CPIII at 10 µM and PPIX at 0.1µM) on co-culture of normal human keratinocytes and melanocytes, using MTT assay after 72 hours of treatment.

(**a**)


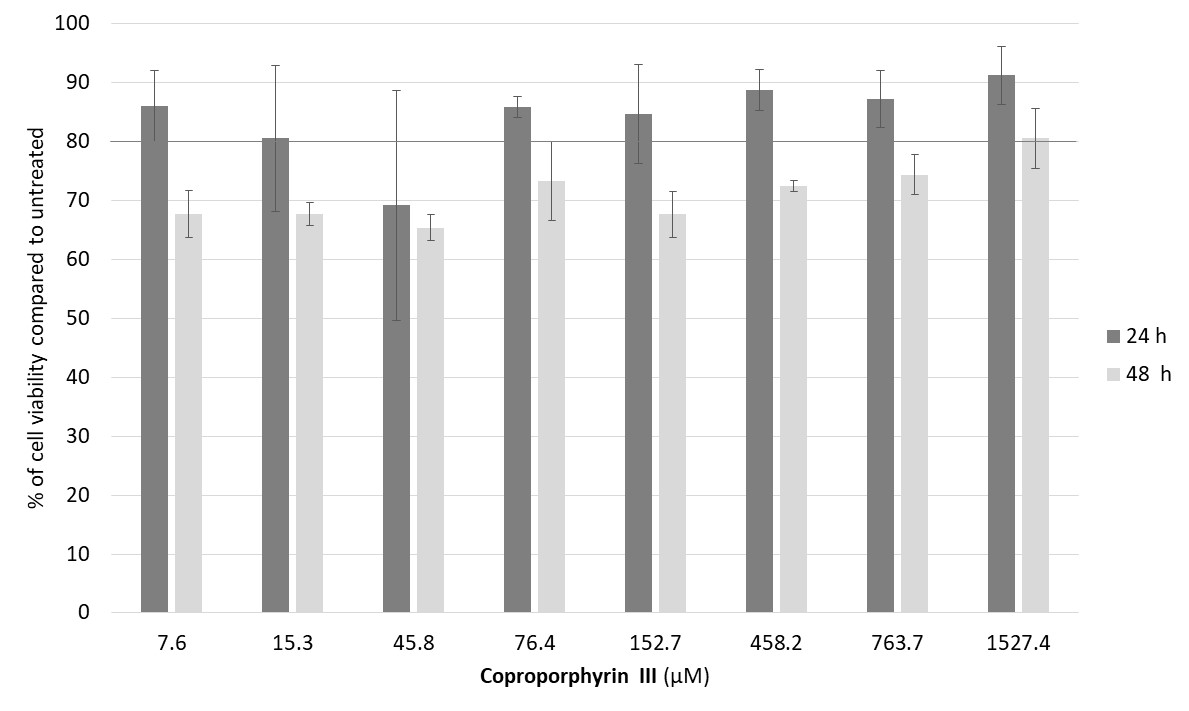


(**b**)


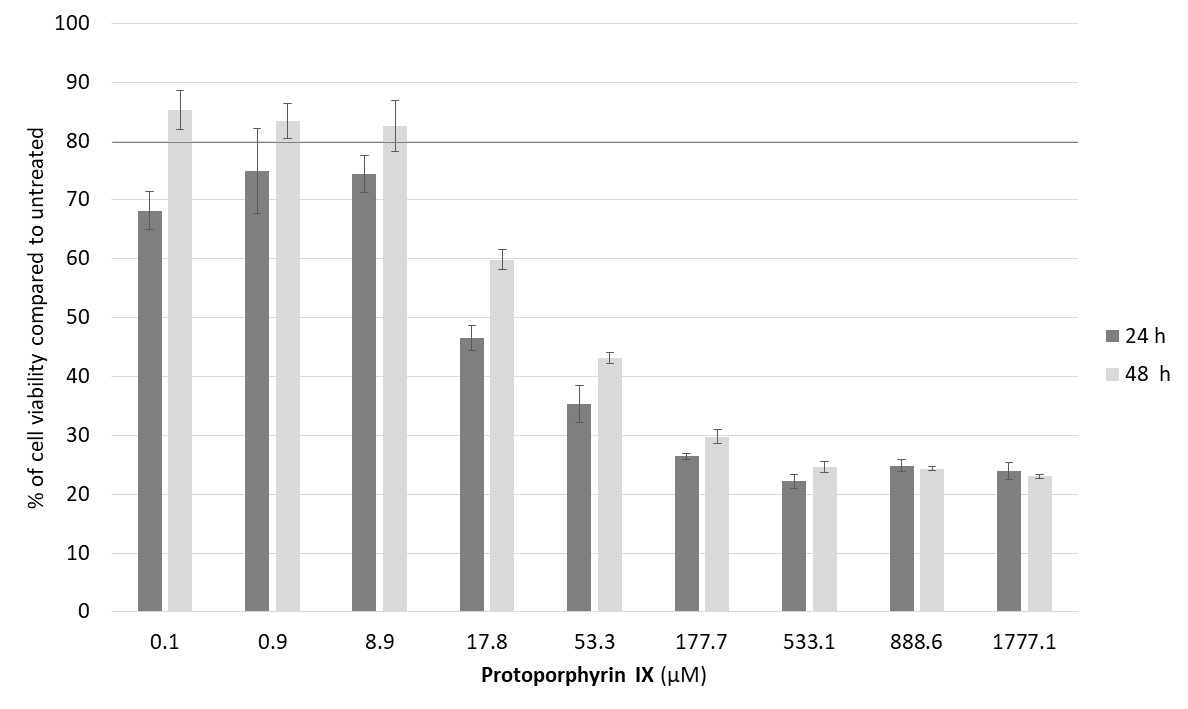


(**c**)


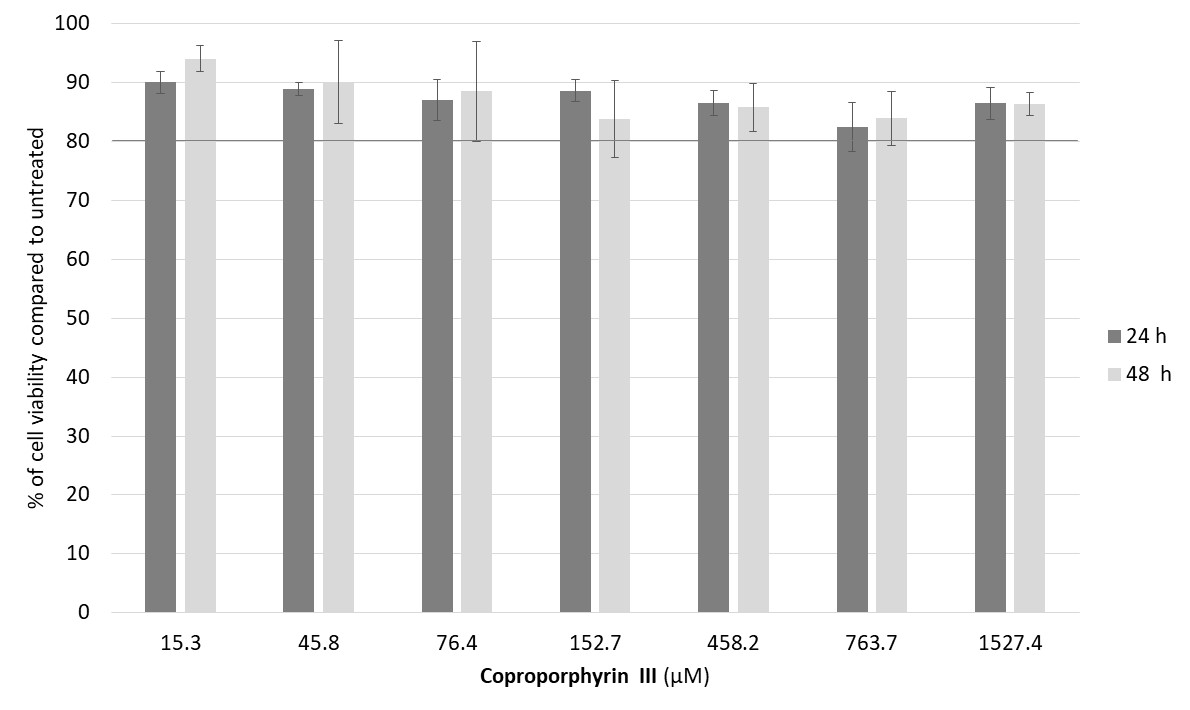


(**d**)


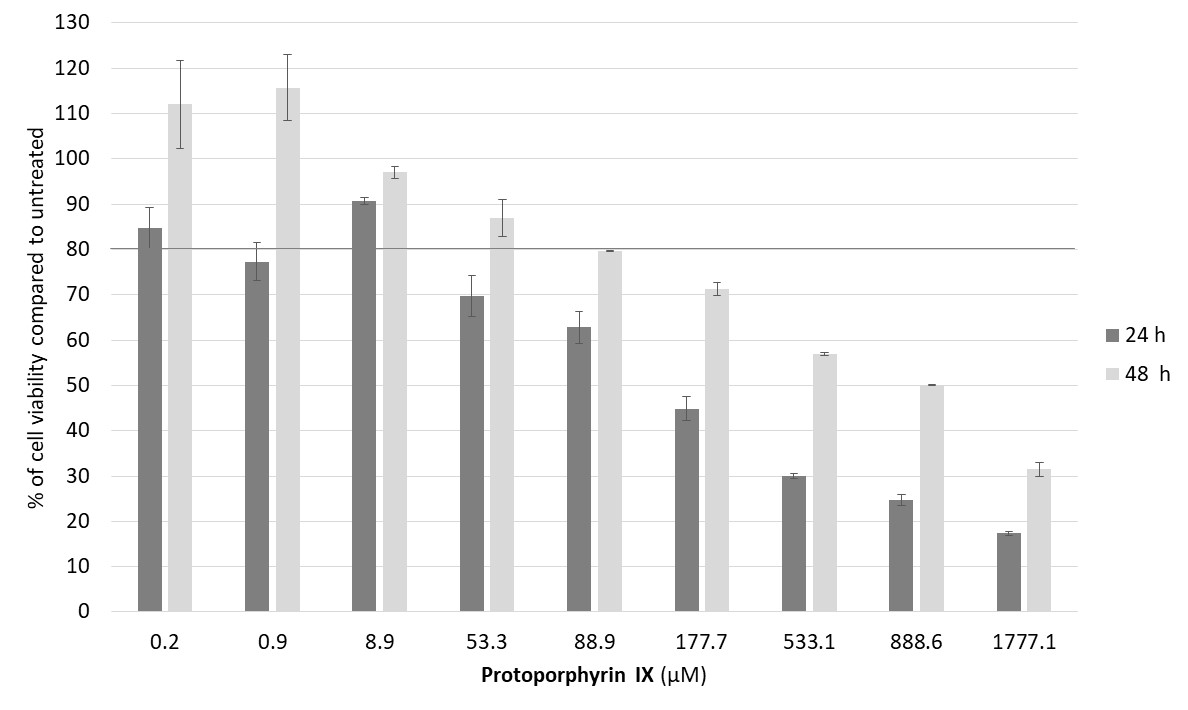


**(e)**
