# Supplementary material for: Bacterial porphyrins in healthy skin: Microbiota components impact melanogenesis and age‐related processes leading to Porphyr'ageing
Source: Int J Cosmet Sci. 2025 Sep 10;48(1):186–99. doi: 10.1111/ics.70014 (PMC12877991; doi:10.1111/ics.70014)
Supplement: Supplementary file 2 — Data S2: [file ICS-48-186-s003.pdf]

Supplementary data S2. Literature review of the role genes down regulated by bacterial porphyrins are playing in skin aging. Concerning *DDIT3*, *LOXL2*, *POU5F*, *MYC*, *BDNF* and *NANOG*, although those genes are involved in cell aging, their role in skin cell aging has not been well characterized yet.
VEGFA: vascular endothelial growth factor A; ***FGF2***: fibroblast growth factor 2; ***IGFBP3***: Insulin-like growth factor-binding protein 3; ***COL4A1***: collagen type IV alpha 1 chain; ***COL7A1***: collagen type VII alpha 1 chain; ***DDIT3***: DNA damage inducible transcript 3; ***GADD45A***: growth arrest and DNA damage inducible alpha; ***XPC***: xeroderma pigmentosum C complex subunit, DNA damage recognition and repair factor; ***GPX1***: glutathione peroxidase 1; ***SOD2***: superoxide dismutase 2; ***COL1A1***: collagen type I alpha 1 chain; ***CTGF***: connective tissue growth factor; ***CYR61***: cysteine rich angiogenic inducer 61 (also called *CCN1*: cellular communication network 1); ***ELN***: elastin; ***FBLN5***: fibulin 5; ***FBN1***: fibrillin 1; ***FBN2***: fibrillin 2; ***FMOD***: fibromodulin; **HPSE**: heparinase; ***HSPG2***: heparan sulfate proteoglycan 2; ***LOXL2***: lysyl oxidase like 2; ***MMP3***: matrix metallopeptidase 3; ***SDC1***: syndecan 1; *SPARC*: secreted protein acidic and cysteine rich; ***TIMP2***: TIMP (tissue inhibitors of matrix metalloproteinases) metallopeptidase inhibitor 2; ***VCAN***: versican; ***TGFB1***: transforming growth factor beta 1; ***SIRT2***: sirtuin 2; ***NQO2***: N-ribosyldihydronicotinamide:quinone dehydrogenase 2; ***RORA***: RAR (retinoic acid receptor) related orphan receptor A; ***BDNF***: brain derived neurotrophic factor; ***NGF***: nerve growth factor; ***NTF3***: neurotrophin 3; ***SIRT1***: sirtuin 1; ***NANOG***: nanog homeobox; ***POU5F1***: POU (Pit-1 Oct-1 Unc-86) class 5 homeobox 1; ***TERT***: telomerase reverse transcriptase; ***MYC***: MYC (myelocytomatosis oncogene) proto-oncogene, bHLH transcription factor.

Gene	Role of the corresponding protein	Observation related to skin aging/cell senescence	References
VEGFA	Growth factor stimulating angiogenesis	Stimulates skin rejuvenation	(Keren et al. 2022)
FGF2	Dermis remodeling	Stimulates skin rejuvenation	(de Araújo et al. 2019)
IGFBP3	Cell proliferation	Decrease with aging	(Hong and Kim 2018)
COL4A1	Dermal-epidermal junction	Decrease in senescent fibroblasts	(Lago and Puzzi 2019)
COL7A1	Dermal-epidermal junction	Decrease in senescent fibroblasts Involved in photo-aging	(Kon et al. 2005; Lago and Puzzi 2019)
DDIT3	DNA damage response	-	-
GADD45A	Cell regulation	Inhibition reduces oxidative stress-induced senescence in fibroblasts	(Haonan et al. 2023)
XPC	DNA repair	Depletion causes premature skin aging in mice	(Hosseini et al. 2015)
GPX1	Antioxidant defense	Potent target to reduce skin aging	(Pourzand et al. 2022)
SOD2	Antioxidant defense	Deficiency impairs mitochondrial activity and induce cell senescence	(Velarde et al. 2012)
COL1A1	Component of ECM	Decrease in skin with aging<br>Decrease in senescent/aged fibroblasts	(Chung et al. 2001; McCabe et al. 2020; Quan et al. 2010)<br>(Bigot et al. 2012; Lago and Puzzi 2019; Waldera Lupa et al. 2015)
CTGF	ECM production	Decrease in skin with aging	(Quan et al. 2010)
CYR61	ECM homeostasis	Overexpressed in aged skin	(Quan et al. 2006)
ELN	ECM component	Enzymatic degradation of elastin fibers during aging<br>Down regulated with aging	(Mora Huertas et al. 2016)<br>(Kim et al. 2015)
FBLN5	ECM component	Down regulated with aging	(Kadoya et al. 2005; McCabe et al. 2020; Zheng et al. 2013)

FBN1	ECM component	Decrease of transcripts with aging	(Kim et al. 2015; Zheng et al. 2013)
FBN2	ECM component	Decrease in aged fibroblast and skin	(McCabe et al. 2020; Waldera Lupa et al. 2015)
FMOD	ECM component	Decrease in senescent fibroblast and skin during aging	(Haga et al. 2024; McCabe et al. 2020)
HPSE	ECM modeling and degradation	Induced by UV-B exposure	(Amano 2016)
HSPG2	ECM component	Decrease in aged skin	(McCabe et al. 2020)
LOXL2	ECM production	-	-
MMP3	ECM degradation	Up regulated in aged fibroblast	(Waldera Lupa et al. 2015)
SDC1	ECM component	Decrease with aging	(Pauly et al. 2009)
SPARC	ECM production and homeostasis	Decrease in aged skin	(Ham et al. 2023)
TIMP2	Regulation of ECM degrading enzymes	Down regulated or up regulated depending on aged fibroblast model	(Lago and Puzzi 2019)
VCAN	ECM component and regulator	Decrease is associated with hair follicle aging	(Jo et al. 2016)
TGFB1	Cell regulation	Regulator of skin aging	(Haga et al. 2024)
SIRT2	Pleitropic effects, gene silencing	Involved in fibroblast senescence	(Anwar et al. 2016; Gilbert et al. 2023)
NQO2	Melatonin receptor	Melatonin protects skin from aging	(Rusanova et al. 2019)
RORA			
BDNF	Growth factor	-	-
NGF	Growth factor	Decrease expression in aged skin	(Adly et al. 2006)
NTF3	Growth factor	Decrease expression in aged skin	(Adly et al. 2017)
SIRT1	Pleiotropic effects, p53 regulator	Age-dependent decrease in vivo and down regulation in senescent fibroblasts	(Bielach-Bazyluk et al. 2021)
NANOG	Cell pluripotency	-	-
POU5F1	Cell pluripotency	-	-
TERT	Telomer synthesis Antioxidant defense	Decrease of telomerase activity in senescent cell	(Bulbiankova et al. 2023; Jacczak et al. 2021)
MYC	Transcription factor	-	-

REFERENCES

Adly MA, Assaf H, Hussein MR. Age-associated decrease of the nerve growth factor protein expression in the human skin: Preliminary findings. Journal of Dermatological Science. Elsevier; 2006;42(3):268–71

Adly MA, Assaf H, Hussein MR. Neurotrophins and Skin Aging. In: Farage MA, Miller KW, Maibach HI, editors. Textbook of Aging Skin. Berlin, Heidelberg: Springer; 2017 [cited 2024 Oct 28]. p. 515–27 Available from: https://doi.org/10.1007/978-3-662-47398-6_15

Amano S. Characterization and mechanisms of photoageing-related changes in skin. Damages of basement membrane and dermal structures. Exp Dermatol. 2016;25 Suppl 3:14–9

Anwar T, Khosla S, Ramakrishna G. Increased expression of SIRT2 is a novel marker of cellular senescence and is dependent on wild type p53 status. Cell Cycle. Taylor & Francis; 2016;15(14):1883–97

Bielach-Bazyluk A, Zbroch E, Mysliwiec H, Rydzewska-Rosolowska A, Kakareko K, Flisiak I, et al. Sirtuin 1 and Skin: Implications in Intrinsic and Extrinsic Aging—A Systematic Review. Cells. Multidisciplinary Digital Publishing Institute; 2021;10(4):813

Bigot N, Beauchef G, Hervieu M, Oddos T, Demoor M, Boumediene K, et al. NF-κB Accumulation Associated with *COL1A1 Trans*activators Defects during Chronological Aging Represses Type I Collagen Expression through a –112/–61-bp Region of the *COL1A1* Promoter in Human Skin Fibroblasts. Journal of Investigative Dermatology. 2012;132(10):2360–7

Bulbiankova D, Díaz-Puertas R, Álvarez-Martínez FJ, Herranz-López M, Barrajón-Catalán E, Micol V. Hallmarks and Biomarkers of Skin Senescence: An Updated Review of Skin Senotherapeutics. Antioxidants. Multidisciplinary Digital Publishing Institute; 2023;12(2):444

Chung JH, Seo JY, Choi HR, Lee MK, Youn CS, Rhie G, et al. Modulation of Skin Collagen Metabolism in Aged and Photoaged Human Skin *In Vivo*. Journal of Investigative Dermatology. 2001;117(5):1218–24

de Araújo R, Lôbo M, Trindade K, Silva DF, Pereira N. Fibroblast Growth Factors: A Controlling Mechanism of Skin Aging. Skin Pharmacology and Physiology. 2019;32(5):275–82

Gilbert MM, Mathes SC, Mahajan AS, Rohan CA, Travers JB, Thyagarajan A. The role of sirtuins in dermal fibroblast function. Front. Med. Frontiers; 2023;10 Available from: https://www.frontiersin.org/journals/medicine/articles/10.3389/fmed.2023.1021908/full

Haga M, Iida K, Okada M. Positive and negative feedback regulation of the TGF-β1 explains two equilibrium states in skin aging. iScience. Elsevier; 2024;27(5) Available from: https://www.cell.com/iscience/abstract/S2589-0042(24)00930-1

Ham SM, Song MJ, Yoon H-S, Lee DH, Chung JH, Lee S-T. SPARC Is Highly Expressed in Young Skin and Promotes Extracellular Matrix Integrity in Fibroblasts via the TGF-β Signaling Pathway. International Journal of Molecular Sciences. Multidisciplinary Digital Publishing Institute; 2023;24(15):12179

Haonan L, Zehang S, Jiacong H, Zhenxing W, Shengli Z, Bailing C, et al. Interleukin-23 mediates the reduction of GADD45a expression to attenuate oxidative stress-induced cellular senescence in human fibroblasts. Mechanisms of Ageing and Development. 2023;212:111808

Hong S, Kim M-M. IGFBP-3 plays an important role in senescence as an aging marker. Environmental Toxicology and Pharmacology. 2018;59:138–45

Hosseini M, Mahfouf W, Serrano-Sanchez M, Raad H, Harfouche G, Bonneu M, et al. Premature Skin Aging Features Rescued by Inhibition of NADPH Oxidase Activity in XPC-Deficient Mice. Journal of Investigative Dermatology. 2015;135(4):1108–18

Jacczak B, Rubiś B, Totoń E. Potential of Naturally Derived Compounds in Telomerase and Telomere Modulation in Skin Senescence and Aging. Int J Mol Sci. 2021;22(12):6381

Jo SJ, Kim JY, Jang S, Choi SJ, Kim KH, Kwon O. Decrease of versican levels in the follicular dermal papilla is a remarkable aging-associated change of human hair follicles. Journal of Dermatological Science. Elsevier; 2016;84(3):354–7

Kadoya K, Sasaki T, Kostka G, Timpl R, Matsuzaki K, Kumagai N, et al. Fibulin-5 deposition in human skin: decrease with ageing and ultraviolet B exposure and increase in solar elastosis. British Journal of Dermatology. 2005;153(3):607–12

Keren A, Bertolini M, Keren Y, Ullmann Y, Paus R, Gilhar A. Human organ rejuvenation by VEGF-A: Lessons from the skin. Science Advances. American Association for the Advancement of Science; 2022;8(25):eabm6756

Kim KS, Park H-K, Lee J-W, Kim YI, Shin MK. Investigate correlation between mechanical property and aging biomarker in passaged human dermal fibroblasts. Microscopy Research and Technique. 2015;78(4):277–82

Kon A, Takeda H, Ito N, Hanada K, Takagaki K. Tissue-specific downregulation of type VII collagen gene (COL7A1) transcription in cultured epidermal keratinocytes by ultraviolet A radiation (UVA) and UVA-inducible cytokines, with special reference to cutaneous photoaging. Journal of Dermatological Science Supplement. 2005;1(2):S29–35

Lago JC, Puzzi MB. The effect of aging in primary human dermal fibroblasts. PLOS ONE. Public Library of Science; 2019;14(7):e0219165

McCabe MC, Hill RC, Calderone K, Cui Y, Yan Y, Quan T, et al. Alterations in extracellular matrix composition during aging and photoaging of the skin. Matrix Biol Plus. 2020;8:100041

Mora Huertas AC, Schmelzer CEH, Hoehenwarter W, Heyroth F, Heinz A. Molecular-level insights into aging processes of skin elastin. Biochimie. 2016;128–129:163–73

Pauly G, Contet-Audonneau J-L, Moussou P, Danoux L, Bardey V, Freis O, et al. Small proteoglycans in the skin: new targets in the fight against skin aging. International Journal of Cosmetic Science. 2009;31(2):154–154

Pourzand C, Albieri-Borges A, Raczek NN. Shedding a New Light on Skin Aging, Iron- and Redox-Homeostasis and Emerging Natural Antioxidants. Antioxidants. Multidisciplinary Digital Publishing Institute; 2022;11(3):471

Quan T, He T, Shao Y, Lin L, Kang S, Voorhees JJ, et al. Elevated Cysteine-Rich 61 Mediates Aberrant Collagen Homeostasis in Chronologically Aged and Photoaged Human Skin. The American Journal of Pathology. 2006;169(2):482–90

Quan T, Shao Y, He T, Voorhees JJ, Fisher GJ. Reduced Expression of Connective Tissue Growth Factor (CTGF/CCN2) Mediates Collagen Loss in Chronologically Aged Human Skin. Journal of Investigative Dermatology. 2010;130(2):415–24

Rusanova I, Martínez-Ruiz L, Florido J, Rodríguez-Santana C, Guerra-Librero A, Acuña-Castroviejo D, et al. Protective Effects of Melatonin on the Skin: Future Perspectives. International Journal of Molecular Sciences. Multidisciplinary Digital Publishing Institute; 2019;20(19):4948

Velarde MC, Flynn JM, Day NU, Melov S, Campisi J. Mitochondrial oxidative stress caused by Sod2 deficiency promotes cellular senescence and aging phenotypes in the skin. Aging (Albany NY). 2012;4(1):3

Waldera Lupa DM, Kalfalah F, Safferling K, Boukamp P, Poschmann G, Volpi E, et al. Characterization of Skin Aging–Associated Secreted Proteins (SAASP) Produced by Dermal Fibroblasts Isolated from Intrinsically Aged Human Skin. Journal of Investigative Dermatology. 2015;135(8):1954–68

Zheng Q, Chen S, Chen Y, Lyga J, Wyborksi R, Santhanam U. Investigation of age-related decline of microfibril-associated glycoprotein-1 in human skin through immunohistochemistry study. CCID. 2013;317
