# Supplementary material for: Bacterial porphyrins in healthy skin: Microbiota components impact melanogenesis and age‐related processes leading to Porphyr'ageing
Source: Int J Cosmet Sci. 2025 Sep 10;48(1):186–99. doi: 10.1111/ics.70014 (PMC12877991; doi:10.1111/ics.70014)
Supplement: Supplementary file 3 — Data S3: [file ICS-48-186-s001.pdf]

Supplementary data S3. Materials and Methods details

Study of bacterial porphyrins effect on NHDFs through gene expression analysis

RNA extraction

Briefly, 1 mL of TRIzol (Thermo Fisher Scientific, Waltham, Massachussets, USA – Carbolic acid, Ammonium thiocyanate, Thiocyanic acid, compound with guanidine (1:1)) was added per well. Cell lysate was transferred in a 2 mL tube, vortexed vigorously for 30 s and incubated at room temperature (RT) for 5 min. A volume of 200 µL of Chloroform:IAA mixture (24:1) (Sigma Aldrich, Saint Louis, Missouri, USA – Chloroform, 3-Methylbutan-1-ol) was added to each tube, which were shaken vigorously 3 times for 10 s. Tubes were incubated at RT for 3 min and were then centrifuged at 12000g for 15 min at 4°C. The aqueous superior phase was collected in a new tube and 500 µL of isopropanol (Sigma Aldrich, Saint Louis, Missouri, USA) was added (which allowed the precipitation of RNA) and mixed by gently inversing. Tubes were incubated at RT for 10 min and then centrifuged at 12000 g for 10 min at 4°C. Supernatants were eliminated and the RNA pellet was then washed with 1 mL Ethanol 75%. Tubes were centrifuged again at 7500 g for 5 min at 4°C. The RNA pellet was dried and dissolved in ultra-pure water. Quantity of total extracted RNA was quantified by spectroscopy (SpectraMax® QuickDrop™, VWR International, Fontenay-sous-Bois, France). All concentrations were adjusted at 250 ng/µL for each condition and the quality of RNA was determined using 4150 TapeStation system (Agilent).

RNA retro-transcription

Reverse transcription was performed to obtain cDNA using Verso cDNA kit and manufacturer instructions (Thermo Fisher Scientific, Waltham, Massachussets, USA – 5X RT Buffer, Random Hexamers, Anchored Oligo dT, 20mM dNTPs, RT enhancer and RT Enzyme mix with RNase inhibitor). A conventional PCR was made in order to check the success of retro-transcription using β-actin amplification by SimpliAmp Thermal Cycler (Applied Biosystem,

Foster City, California, USA). The human β-actin (Forward: 5'-GTGTGACGTGGACATCCGC-3'; Reverse: 5'-CTGCATCCTGTCGGCAATG-3') primers were used with the following PCR program: 1) 94°C for 3 min to activate Taq enzyme; 2) cDNA strands denaturation at 94°C for 30 seconds; 3) primers hybridation at 60°C for 30 s; 4) elongation step at 72°C for 30 s; 5) 30 repetitions of steps 2-3-4; 6) final elongation at 72°C for 10 min.

qPCR on SYBR Green plates

The cDNAs were concentrated at 1000 ng in 40 µl. A volume of 960µl of ultra-pure water was added to obtain a final concentration of 1 ng.μL^{-1} of cDNA. 10µL of cDNA (10 ng) were first deposed per well in specific designed plate and completed with 10 µL of iTaq Universal Sybr Green supermix (Bio-RAD). The RT-qPCR was performed using CFX96 Touch (Bio-Rad). The relative quantification (RQ) of gene expression was calculated according to two housekeeping genes.
